# The Oncoplacental Gene Placenta-Specific Protein 1 Is Highly Expressed in Endometrial Tumors and Cell Lines

**DOI:** 10.1155/2013/807849

**Published:** 2013-07-10

**Authors:** Eric J. Devor, Kimberly K. Leslie

**Affiliations:** Department of Obstetrics and Gynecology, University of Iowa Carver College of Medicine, 3234 MERF, 375 Newton Road, Iowa City, IA 52242, USA

## Abstract

Placenta-specific protein 1 (PLAC1) is a small secreted protein expressed exclusively in trophoblast cells in the mammalian placenta. PLAC1 is expressed early in gestation and is maintained throughout. It is thought to function in trophoblast invasion of the uterine epithelium and, subsequently, to anchor the placenta to the epithelium. In recent years, evidence has accumulated that PLAC1 is also expressed in a variety of human solid tumors, notably in breast cancers. We demonstrate for the first time that PLAC1 is ubiquitously expressed in tumors originating in uterine epithelium. Further, we find that PLAC1 expression is significantly higher in the more advanced, more aggressive endometrial serous adenocarcinomas and carcinosarcomas relative to endometrioid adenocarcinomas by more than 6-fold and 16-fold, respectively. We also show that PLAC1 is simultaneously transcribed from two promoters but that, in all cases, the more distal P1 promoter dominates the more proximal P2 promoter. While the function of the two PLAC1 promoters and their regulation are as yet unknown, overall expression data suggest that PLAC1 may serve as a biomarker for endometrial cancer as well as a potential prognostic indicator.

## 1. Introduction

Placenta-specific protein 1 (PLAC1), encoded on human chromosome Xq26, is a small (212 amino acid) secreted protein whose normal expression is almost exclusively limited to placental trophoblast cells [1, 2]. Comparative genomics reveals that PLAC1 evolved after the divergence of placental mammals (Eutheria) from marsupials (Metatheria) as there are homologs throughout the former but no evidence in either of the other mammalian subclasses Metatheria and Prototheria (egg laying mammals) [3]. Among all Eutheria so far studied, PLAC1 shows a well conserved signal peptide (residues 1–23), a very highly conserved transmembrane domain (TMD) (residues 20–50), and a highly conserved region in the extracellular domain homologous to the N-terminal subdomain of the zona pellucida ZP3 glycoprotein (residues 58–118) [4, 5]. Normal expression of the PLAC1 protein is limited to the apical villous surface of syncytiotrophoblasts suggesting that it is involved in anchoring the placenta to the endometrium and maintaining that contact throughout gestation [2]. Moreover, the ZP3-like extracellular domain suggests that strong protein binding interactions are likely [6], and evidence that PLAC1 and F-actin colocalize further supports this view [2].

In addition to the highly specific expression in normal placental development and maintenance, several studies have detected strong PLAC1 expression in a number of human solid tumors prompting the classification of PLAC1 as an oncoplacental protein [7], a class of protein in which PLAC1 remains the sole member. Among the tumors where PLAC1 expression has been detected are nonsmall cell lung cancers [8], breast cancers [5], hepatocellular and colorectal cancers [9, 10], and gastric cancers [11]. In addition, PLAC1 expression has been demonstrated in nearly one hundred cancer cell lines representing fourteen different cancers [5, 8, 9]. However, to date, PLAC 1 expression has not been reported in endometrial cancers. Here, we demonstrate ubiquitous PLAC1 expression in a panel of endometrial tumors as well as in endometrial cancer cell lines. Moreover, we show that PLAC1 expression is significantly greater in the higher stage, more aggressive uterine serous adenocarcinomas and carcinosarcomas.

## 2. Materials and Methods

### 2.1. Study Subjects, Tissue Collection, and RNA Preparation

The endometrial tissue panel used in this study is composed of four benign endometrium tissues, nine endometrioid adenocarcinomas, eight serous adenocarcinomas, and seven endometrial carcinosarcomas (Table 1(a)). All tissues were obtained under informed consent, and with IRB approvals, from patients undergoing surgery at the University of Iowa Hospitals and Clinics. Endometrial cancer cell lines used were Ishikawa-H, ECC-1, KLE, RL95-2, KLE, Hec50co, An3CA, and SK-UT-1b (Table 1(b)). All cell lines were grown under optimum conditions, and cells were harvested for RNA preparation at 80% to 90% confluence.

Total cellular RNAs were purified from flash frozen tumor tissue samples and harvested cultured cells using the miRvana RNA isolation kit according to manufacturer's instructions (Ambion, Life Technologies). RNA yield and quality was determined using a NanoDrop M-1000 spectrophotometer and an Agilent 2100 Bioanalyzer. Acceptable RNA quality were assigned to RNAs having RIN ≥ 7.00, and those RNAs were standardized to 100 ng/*μ*L for subsequent expression assays.

### 2.2. PCR and qPCR Primer Design

**Figure 1:**
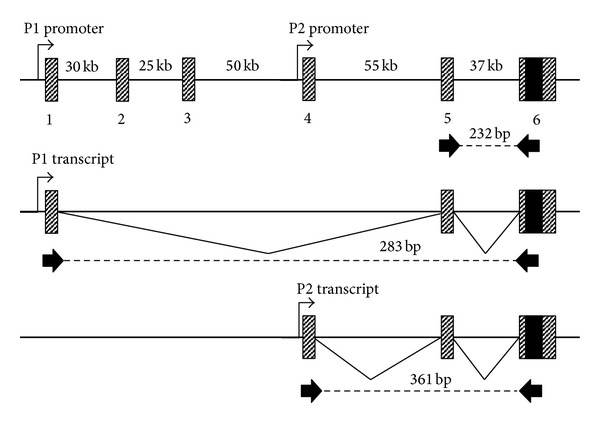
Genomic organization of the human PLAC1 gene on chromosome Xq26. The five 5′ UTR exons and the 5′ UTR and 3′ UTR components of Exon 6 are cross-hatched. The protein coding region in Exon 6 is the solid box. The P1 and P2 promoters are indicated relative to Exons 1 and 4 along with the composition of the P1 and P2 transcripts. Locations of the RT-PCR/qPCR primers in Table 2 are indicated along with their amplicon sizes.

PLAC1 genomic organization is presented in Figure 1. The gene is composed of six exons, of which five constitute a series of alternately spliced 5′ UTRs [19]. The 3′ end of the 5′ UTR, the entire coding region, and the 3′ UTR are all contained within the 898 bp long Exon 6. Two 5′ UTR variants dominate PLAC1 expression (Figure 1). One transcript, containing Exons 1-5-6, is transcribed from a promoter termed P1 lying 5′ of Exon 1, while the second transcript, containing Exons 4-5-6, is transcribed from a promoter termed P2 lying in Intron 3 [19, 20]. Using genome sequence information from Ensembl as well as from Chen et al. [19], we designed PCR primers for use in both conventional and quantitative PCR. Primer sequences are shown in Table 2. All PCR primers were purchased from IDT (Integrated DNA Technologies). Primer secondary structure and dimer formation were evaluated using PrimerQuest (IDT). In some instances, PrimerQuest-designed sequences partially overlap previously published sequences [8, 19]. Primer specificity was further evaluated via BLAST.

Prior to qPCR, all primer pairs were validated by conventional PCR and direct sequencing of amplicons.

### 2.3. Reverse Transcription PCR

Reverse transcription was performed on 250 ng aliquots of total RNA from all cell lines and tissue samples using SuperScript III RT (Invitrogen). PLAC1 expression was determined via amplification of cDNA with the Exon 5-6 PCR primers (Table 2). PCR conditions were conventional three-step amplification for 35 cycles at an annealing temperature of 60.0°C.

RT-PCR amplicons were run out on a 1.5% agarose gel in 1X TBE. The expected 232 bp amplicon was confirmed with a 100 bp ladder (Invitrogen Trackit).

### 2.4. Quantitative PCR

Quantitative PLAC1 expression was assessed via SYBR Green qPCR assay. Reverse transcription was performed on 350 ng aliquots of total RNA from endometrial cancer cell lines and both benign and cancerous endometrial tissue samples using SuperScript III RT (Invitrogen). Resulting cDNAs were equally aliquoted into four reactions for qPCR of total PLAC1 message (Exon 5-6 primers), P1 transcribed message (Exon 1-5-6 primers), P2 transcribed message (Exon 4-5-6 primers), and 18S rRNA endogenous control (Table 2). All primer pairs were validated for specificity by conventional gel electrophoresis and dissociation curve.

SYBR Green qPCR amplifications were carried out in triplicate in Power SYBR Green mix (Applied Biosystems, Life Technologies) in a 384-well format on an Applied Biosystems Model 7900 Genetic Analyzer. Cycle thresholds were normalized against 18s rRNA. Fold change was determined via the standard ΔΔCt method [21, 22] and statistical significance assessed by a conventional *t*-test with unequal variances [23].

## 3. Results

### 3.1. PLAC1 Expression in Endometrial Tumors and Cell Lines

**Figure 2:**
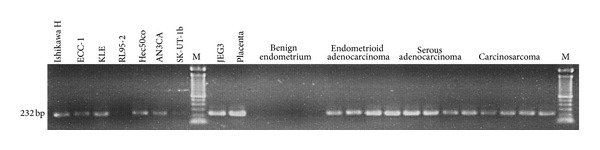
RT-PCR amplification of PLAC1 message in seven endometrial cancer cell lines and representative endometrial tumors (*n* = 4 each). Primers used for this assay are the Exon 5-6 pair (Table 2). JEG3 is a choriocarcinoma cell line known to express PLAC1 [8]. Placental tissue is from a normal 38-week delivery. Molecular weight markers (M) are Invitrogen Trackit 100 bp ladder.

The presence of PLAC1 mRNA transcripts in both endometrial cancer cell lines and endometrial tumors is seen in Figure 2. PCR amplification was carried out using the Exon 5-6 primer pair that produces a 232 bp amplicon from cDNA (Table 2). Consistent with the known PLAC1 expression pattern, none of the four benign endometrium samples produced PLAC1 amplicons though all four did produce 18S rRNA amplicons. PLAC1 transcript was detected to varying degrees among the seven endometrial cancer cell lines. RNAs from the choriocarcinoma cell line JEG3, known to express PLAC1 [8], and from a 38-week human placenta were included as positive controls. The three major endometrial cancer tumor types, endometrioid adenocarcinoma, serous adenocarcinoma, and carcinosarcoma, are represented by four samples each. These were selected from the full panel of nine endometrioid adenocarcinomas, eight serous adenocarcinomas, and seven carcinosarcomas, all of which produced both PLAC1 and 18S rRNA amplicons.

### 3.2. PLAC1 Quantitative PCR

The SYBR Green qPCR assay of all seven endometrial cancer cell lines and all twenty-four endometrial tumors, using the Exon 5-6 primers, was consistent with the conventional PCR results. Among the endometrial cancer cell lines Ishikawa H and Hec50co cells displayed the highest, nearly equal PLAC1 expression with Hec50co cells being 1.26-fold higher relative to Ishikawa H. KLE cells presented PLAC1 expression of −3.26-fold relative to Ishikawa H. Both ECC-1 and AN3CA cells were more than 80-fold lower than Ishikawa H, but neither RL95-2 nor SK-UT-1b cells presented any appreciable PLAC1 expression relative to the others.

**Figure 3:**
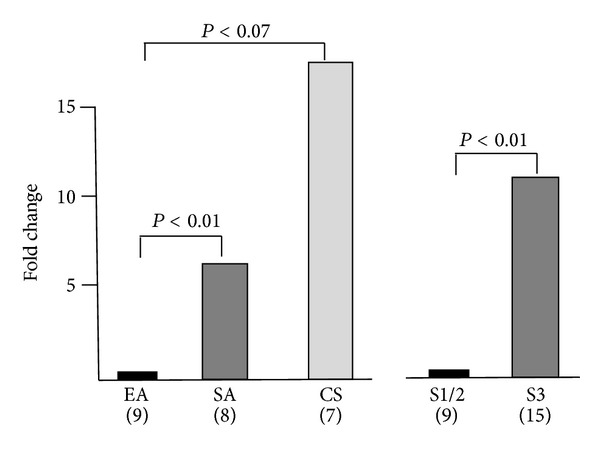
Relative PLAC1 expression among endometrial tumor types (left) and between tumor stages (right). Fold changes, assessed by ΔΔCt, between endometrioid adenocarcinoma (EA) and serous adenocarcinoma (SA) and between endometrioid adenocarcinoma (EA) and carcinosarcoma (CS) are 6.58 and 16.47, respectively. PLAC1 fold change between Stage 1 and 2 tumors (S1/2) and Stage 3 (S3) tumors is 10.41. Statistical significance is assessed by a two-tailed *t*-test with unequal variances using normalized expression values (ΔCt). Sample sizes are shown.

Endometrial tumors showed far less volatility in PLAC1 expression than did the cell lines. However, the higher stage, more aggressive serous adenocarcinomas and carcinosarcomas did display significantly higher PLAC1 expression than the lower stage, less aggressive endometrioid tumors (Figure 3). Serous adenocarcinomas display a 6.6-fold higher PLAC1 expression (*P* < 0.01) relative to endometrioid adenocarcinomas, and carcinosarcomas display a 16.5-fold higher PLAC1 expression (*P* < 0.07) relative to endometrioid adenocarcinomas. When tumor stage is considered, Stage 3 tumors display 10.41-fold higher PLAC1 expression than do Stage 1 and 2 tumors (*P* < 0.01). However, this result is essentially redundant as there were no Stage 3 endometrioid tumors in our sample nor were there any Stage 1 or Stage 2 serous adenocarcinomas or carcinosarcomas (see Table 1). The importance of stage versus tumor type cannot be determined until PLAC1 expression is determined in higher stage endometrioid adenocarcinomas as well as lower stage serous adenocarcinomas and carcinosarcomas.

### 3.3. Promoter-Specific qPCR

**Figure 4:**
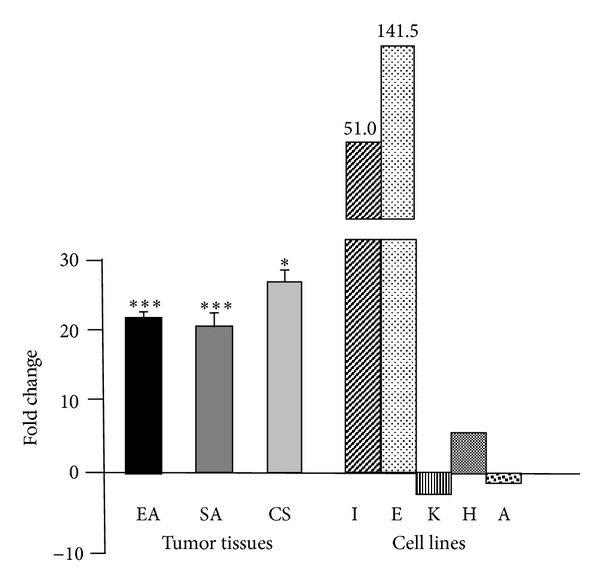
Relative expression of P1 transcripts and P2 transcripts in both endometrial tumors and cultured cell lines. Fold change of P1 transcript versus P2 transcript in each tumor type, assessed by ΔΔCt, was calculated for each individual sample and then averaged. Statistical significance is assessed by a two-tailed *t*-test with unequal variances using normalized expression values (ΔCt). EA is endometrioid adenocarcinoma, SA is serous adenocarcinoma, and CS is carcinosarcoma. Cell lines are Ishikawa H (I), ECC-1 (E), KLE (K), Hec50co (H), and AN3CA (A). *P < 0.05, ***P < 0.001.

P1 and P2 transcript-specific SYBR Green assays showed that P1 transcribed message is significantly more abundant than P2 transcribed message in all twenty-four tumors. In the endometrioid adenocarcinomas P1 transcript abundance was 22.1-fold higher than P2 transcript abundance (*P* < 0.001), in the serous adenocarcinomas P1 transcript abundance was 20.3-fold higher than P2 transcript abundance (*P* < 0.001), and in carcinosarcomas P1 transcript abundance was 28.8-fold higher than P2 transcript abundance (*P* < 0.05) (Figure 4). There was a considerable range of P1 transcript abundance from a low of 2.1-fold in one of the carcinosarcomas to a high of 430.2-fold in a serous adenocarcinoma. Though no individual tumor showed an overabundance of P2 transcript, two of the five cell lines, KLE and AN3CA, did (Figure 4). Such variation in relative transcript abundance is consistent with other cultured cell line data [19], but, as this is the first time similar data have been collected from individual tumors of any kind, it is unknown if other tumors display similar variation.

## 4. Discussion

We have shown both through conventional RT-PCR and SYBR Green qPCR assays that the gene encoding the oncoplacental protein PLAC1 is expressed in endometrial cancer cell lines and in all three of the most common endometrial tumors. These results add to the growing list of human solid tumors in which PLAC1 expression has been demonstrated [5, 8–10]. It is important to note that PLAC1 expression is seen in all twenty-four endometrial tumors irrespective of tumor type. Ubiquitous PLAC1 expression has not previously been reported. The closest any cancer has come is 29 of 32 breast cancers (90.6%) [5], while other tumors such as nonsmall cell lung cancer (5 of 8, 62.5%) [8], hepatocellular cancer (32 of 69, 46.4%) [9], and colorectal cancer (22 of 42, 52.4%) [10] present PLAC1 expression much less often. The reason for such nearly universal PLAC1 expression in breast and uterine tumors may be that these cancers are far more hormone sensitive than are the others so far reported, and there is evidence from breast cancers that one of the PLAC1 promoters, P2, is estrogen responsive. In a paired set of ER*α*-positive and ER*α*-negative breast tumors (*n* = 20 each), there was a statistically significant increase in P2 transcript in the ER*α*-positive group [20]. Detailed examination of the P2 promoter in the ER*α*-positive MCF-7 breast cancer cell line demonstrated that ER*α* activates the P2 promoter via a pathway independent of estrogen response elements [20]. The relationship between ER*α* and PLAC1 promoters in tumors has not been explored. However, it is well established that presence or absence of ER*α* in endometrial cancers, often in relation to progesterone receptor and Er*β*, is related to treatment response and survival [24]. Thus, this dimension must also be investigated.

In general, the reason for the presence or absence of PLAC1 expression at all in a tumor is unknown. Several excellent studies of PLAC1 expression in placentae have shown that the protein is exclusively expressed at the apical surface of trophoblasts [2, 4, 25] and that expression begins very early in gestation and remains throughout [26]. Mature PLAC1 protein localizes in the cell membrane with the entire post-TMD, including the highly conserved, potentially reactive ZP3 domain, in the intracellular milieu between placenta and uterine epithelium. This has led to speculation that PLAC1 likely serves to assist in trophoblast invasion of the endometrium and subsequent anchoring of the placenta. Indirect support for this idea comes from experiments in MCF-7 and BT-549 breast cancer cells showing that PLAC1 knockdown significantly reduces cell motility, proliferation, and invasiveness [5]. It seems reasonable to assume that it is these very properties, derived from the gene's normal function, which lead cancer cells to coopt the PLAC1 gene.

In addition to ubiquitous PLAC1 expression in the tumor panel, of the seven endometrial cancer cell lines examined here, only the five cell lines derived from endometrioid adenocarcinomas, Ishikawa H, ECC-1, KLE, Hec50co, and AN3CA, evidenced appreciable PLAC1 expression. Of the two cell lines that failed to express PLAC1 mRNA one, RL95-2, is from a Grade 2 adenosquamous tumor [15], and the other, SK-UT-1b, is from a Grade 3 leiomyosarcoma [18]. It is presently unknown whether or not the origin and type of endometrial cancer triggers PLAC1 expression, but it may not be coincidental that the five endometrial cancer cell lines that do express PLAC1 all originated in uterine epithelium as did, by definition, all seventeen adenocarcinomas in the tumor panel. Further, uterine carcinosarcomas have been shown to originate as monoclonal tumors in uterine epithelium that subsequently differentiate into carcinomatous/epithelial and sarcomatous/mesenchymal components [27]. Whether this warrants a conclusion that tumors arising in uterine epithelium either preferentially or exclusively activate PLAC1 expression must await further study of a range of uterine tumors. 

Another question that must receive further attention is the mechanism of transcription of PLAC1 message both in tumors and placenta. As shown in Figure 1, PLAC1 genomic structure is composed of six exons wherein the 3′-most 58 bp of the 5′ UTR, the entire 639 bp coding region, and the 201 bp 3′ UTR are entirely contained within the sixth exon. The other five exons form several alternately spliced 5′ UTRs [19]. Among these, only two, one composed of Exons 1-5-6 and another composed of Exons 4-5-6, are expressed, but they are expressed simultaneously through different promoters. Exon 4-5-6 mRNA, the P2 transcript, is transcribed from a canonical SP1 site and from an unusual isoform of CCAAT/enhancer-binding protein *β* (C/EBP *β*-2), both of which are located just upstream from Exon 4 and are estrogen responsive [20]. A second promoter, termed P1, is just upstream from Exon 1 [19]. Moreover, mRNA transcribed from both promoters is present in total PLAC1 message [19]. P2-driven transcription accounts for the majority of PLAC1 message in human placenta, but P1-driven transcription accounts for the majority of PLAC1 message in several cancer cell lines. We see from our own promoter-specific PCR and qPCR amplifications that the same holds true for the endometrial cancer cell lines and tumors. Our own data show that P1 accounts for significantly more PLAC1 message in all of the endometrial tumors, but there is a mixture of P1-driven and P2-driven messages in the endometrial cancer cell lines derived from adenocarcinomas. The importance of this dual promoter transcription is not clear. To date, our data are the only data from primary tumors tissues to assess the relative contribution of the two promoters to total PLAC1 message even in a preliminary way.

## 5. Conclusion

Placenta specific protein 1 (PLAC1), which normally is exclusively expressed in placental trophoblasts, is an important element in the establishment and maintenance of the placenta. Several studies have shown that the PLAC1 gene is turned on in a variety of human solid tumors and cancer cell lines. We have shown here that PLAC1 expression appears to be ubiquitous in cancers originating in uterine epithelium. We also demonstrate that total PLAC1 message originates in two promoters simultaneously though the specific mechanism and reason for this remain unknown at the present time. Our study suffers from two weaknesses. First, our sample sizes are small. Second, while we succeeded in purifying high quality RNA from the tissues, we did not obtain protein lysates with which to examine PLAC1 expression at that level. We believe, however, that further detailed study of PLAC1 in endometrial cancers is warranted and will ultimately lead to elucidation of the role of PLAC1 in uterine carcinogenesis, the mechanism determining PLAC1 transcription initiation, the role of estrogen in PLAC1 transcription, and development of PLAC1 as a biomarker of endometrial carcinogenesis and prognosis.

## Figures and Tables

**Table:** (a) Endometrial cancer patient panel

ID no.	Age	Tumor type	Stage	Grade
BE226	45	Benign endometrium		
BE227	46	Benign endometrium		
BE243	32	Benign endometrium		
BE253	43	Benign endometrium		

EA45	71	Endometrioid adenocarcinoma	2	IB
EA54	84	Endometrioid adenocarcinoma	2	IB
EA68	49	Endometrioid adenocarcinoma	2	IIB
EA69	58	Endometrioid adenocarcinoma	1	IB
EA70	62	Endometrioid adenocarcinoma	1	IB
EA74	74	Endometrioid adenocarcinoma	2	IA
EA81	44	Endometrioid adenocarcinoma	1	IA
EA83	85	Endometrioid adenocarcinoma	1	IA
EA115	55	Endometrioid adenocarcinoma	2	IB

SA48	60	Serous adenocarcinoma	3	IVB
SA72	83	Serous adenocarcinoma	3	IIIC
SA79	87	Serous adenocarcinoma	3	IIIC
SA93	85	Serous adenocarcinoma	3	IIIC
SA169	81	Serous adenocarcinoma	3	II
SA178	71	Serous adenocarcinoma	3	IC
SA208	70	Serous adenocarcinoma	3	IA
SA289	83	Serous adenocarcinoma	3	IIIC2

CS5	63	Carcinosarcoma	3	IIIC
CS21	77	Carcinosarcoma	3	IC
CS32	61	Carcinosarcoma	3	IIIC
CS114	47	Carcinosarcoma	3	IIIC
CS335	78	Carcinosarcoma	3	IA
CS352	54	Carcinosarcoma	3	IA
CS355	60	Carcinosarcoma	3	IIIA

**Table:** (b) Endometrial cancer cell lines

Cell line	Age	Tumor type	Source	Reference
Ishikawa H	39	Endometrioid adenocarcinoma	Gift	[12]
ECC-1	68	Endometrioid adenocarcinoma*	ATCC	[13]
KLE	64	Endometrioid adenocarcinoma	ATCC	[14]
RL95-2	65	Adenosquamous carcinoma	ATCC	[15]
Hec50co	na	Endometrioid adenocarcinoma^#^	In-house	[16]
AN3CA	55	Endometrioid adenocarcinoma mets^§^	ATCC	[17]
SK-UT-1b	75	Leiomyosarcoma	ATCC	[18]

*tumor from luminal epithelium.

^#^will produce serous tumors in mouse explants.

^§^associated with a primary diagnosis of acanthosis nigricans.

**Table 2:** Primer sequences used for conventional and quantitative PCR assays.

Amplicon	Size	Sequence	*T*_*m*_
Exon 6 coding region	812 bp	Forward: 5′-TCCTGTTTCCTGTGGTTCATT-3′	62.0°C
		Reverse: 5′-TCATGAAGTTGCTATAGGTTTCTCT-3′	62.0°C

Exon 5-6*	232 bp	Forward: 5′-CACCAGTGAGCACAAAGCCACATT-3′	60.3°C
		Reverse: 5′-CCATGAACCAGTCTATGGAG-3′	52.3°C

Exon 4-5-6^#^	361 bp	Forward: 5′-GTGACTCTCCTATGAAGGTAAAGG-3′	54.4°C
		Reverse: 5′-CCATGAACCAGTCTATGGAG-3′	52.3°C

Exons 1-5-6^§^	283 bp	Forward: 5′-AAACTTACACGAGGAGTCTGTC-3′	57.2°C
		Reverse: 5′-CTGTGACCATGAACCAGTCTAT-3′	54.2°C

18S rRNA	104 bp	Forward: 5′-AACTTTCGATGGTAGTCGCCG-3′	57.3°C
		Reverse: 5′-CCTTGGATGTGGTAGCCGTTT-3′	57.6°C

*qPCR assay for total PLAC1 mRNA.

^#^qPCR assay for Promoter 2 transcribed PLAC1 mRNA.

^§^qPCR assay for Promoter 1 transcribed PLAC1 mRNA.
